# Prognostic Factors in COVID-19 Patients With New Neurological Manifestations: A Retrospective Cohort Study in a Romanian Neurology Department

**DOI:** 10.3389/fnagi.2021.645611

**Published:** 2021-06-17

**Authors:** Eugenia Irene Davidescu, Irina Odajiu, Delia Tulbǎ, Constantin Dragoş Sandu, Teodora Bunea, Georgiana Sandu, Dafin Fior Mureşanu, Paul Bǎlǎnescu, Bogdan Ovidiu Popescu

**Affiliations:** ^1^Neurology Department, Colentina Clinical Hospital, Bucharest, Romania; ^2^Department of Clinical Neurosciences, “Carol Davila” University of Medicine and Pharmacy, Bucharest, Romania; ^3^Colentina—Research and Development Center, Colentina Clinical Hospital, Bucharest, Romania; ^4^Department of Neurosciences, “Iuliu Haţieganu” University of Medicine and Pharmacy, Cluj-Napoca, Romania; ^5^“Carol Davila” University of Medicine and Pharmacy, Bucharest, Romania; ^6^Clinical Research Unit RECIF (Reseau d'Epidemiologie Clinique International Francophone), Bucharest, Romania; ^7^Laboratory of Cell Biology, Neurosciences and Experimental Myology, “Victor Babeş” National Institute of Pathology, Bucharest, Romania

**Keywords:** COVID-19, elderly, neurological symptoms, risk factors, confusion, stroke, headache, mortality

## Abstract

**Introduction:** The emerging Coronavirus Disease (COVID-19) pandemic caused by Severe acute respiratory syndrome coronavirus 2 (SARS-CoV-2) is a serious public health issue due to its rapid spreading, high mortality rate and lack of specific treatment. Given its unpredictable clinical course, risk assessment, and stratification for severity of COVID-19 are required. Apart from serving as admission criteria, prognostic factors might guide future therapeutic strategies.

**Aim:** We aimed to compare clinical features and biological parameters between elderly (age ≥ 65 years) and non-elderly (age <65 years) patients with COVID-19 and new neurological symptoms/conditions. We also aimed to determine factors independently associated with all-cause in-hospital mortality.

**Methods:** All consecutive patients with COVID-19 and new neurological symptoms/conditions admitted in our Neurology Department between April 1 and August 23, 2020 were enrolled in this observational retrospective cohort study. Patient characteristics such as demographic data, comorbidities, biological parameters, imaging findings and clinical course were recorded. All-cause in-hospital mortality was the main outcome, whereas COVID-19 severity, hospitalization duration and the levels of supplemental oxygen were the secondary outcomes.

**Results:** One hundred forty-eight patients were included, out of which 54.1% were women. The average age was 59.84 ± 19.06 years and 47.3% were elderly, the majority having cardiovascular and metabolic comorbidities. In the elderly group, the most frequent neurological symptoms/manifestations responsible for hospitalization were stroke symptoms followed by confusion, whereas in the non-elderly, headache prevailed. The final neurological diagnosis significantly varied between the two groups, with acute cerebrovascular events and acute confusional state in dementia most commonly encountered in the elderly (65.71 and 14.28%, respectively) and secondary headache attributed to SARS-CoV-2 infection often experienced by the non-elderly (38.46%). The elderly had statistically significant higher median values of white blood cell (8,060 vs. 6,090/μL) and neutrophil count (6,060 vs. 4,125/μL), C-reactive protein (29.2 vs. 5.72 mg/L), ferritin (482 vs. 187 mg/dL), fibrinogen (477 vs. 374 mg/dL), D-dimer (1.16 vs. 0.42), prothrombin time (151.15 vs. 13.8/s), aspartate transaminase (26.8 vs. 20.8 U/l), creatinine (0.96 vs. 0.77 mg/dL), and blood urea nitrogen level (51.1 vs. 27.65 mg/dL), as well as lower median value of hemoglobin (13.05 vs. 13.9 g/dL) and lymphocyte count (1,245 vs. 1,670/μL). Moreover, advanced age was significantly associated with more extensive lung involvement (25 vs. 10%) and higher fatality rate (40 vs. 9%). Overall, the mortality rate was 23.6%. Age as well as neutrophil count, C-reactive protein, fibrinogen, and activated partial thromboplastin time levels were independently associated with mortality.

**Conclusions:** Older age, higher neutrophil count, C-reactive protein, fibrinogen, and activated partial thromboplastin time levels are independent predictors of mortality in COVID-19 patients with new neurological manifestations/conditions at admission.

## Introduction

The current pandemic of Severe Acute Respiratory Syndrome Coronavirus 2 (SARS-CoV-2) responsible for Coronavirus Disease 2019 (COVID-19) has rapidly led to significant changes in all aspects of our existence, mainly due to its large-scale spreading, high mortality rate, and lack of specific treatment.

The elderly patients are the most affected by COVID-19, since the mortality rate gradually increases with age. Fatality rate differs between countries; in addition to the epidemiological evolution of the pandemic, it is related to different demographic and socio-economic aspects. Considering the higher life-expectancy in high-income countries, the mortality rate due to COVID-19 in people over 70 years reaches 87%, whereas in low-income countries it is around 37% (Demombynes, 2020). For instance, in the United States more than 80% of deaths amid adults occurred in patients aged ≥65 years (Centers for Disease Control Prevention., 2020) whereas in Romania a similar rate was registered in patients older than 60 years (Institutul National de Sănătate Publică, 2020). In Germany, it was estimated that the lifespan of patients with multiple comorbidities is reduced by 9 years due to SARS-CoV-2 infection (Deutsche Akademie der Naturforscher Leopoldina e.V., 2020). Another important indicator is the excess mortality that shows higher numbers for older subjects especially in the European population—more than 30,000 excessive deaths for people over 65 years during the first wave of the pandemic in comparison to around 2,500 deaths for subjects between 45 and 64 years old (Excess Mortality., 2020a). The situation in the United States is slightly different, since there is an excess mortality of 14.4% for adults aged 45–64, 24.1% for 65–74 years, 21.4% for 75–84 years, and 14.7% for ≥85 years old (Excess Mortality., 2020b). Gender differences were also noticed, with males experiencing more aggressive forms of SARS-CoV-2 infection as well as higher mortality rates with aging compared to females (Karlberg et al., 2004).

Neurologic manifestations seem to be encountered rather frequently in COVID-19 patients, ranging from 36.4% of hospitalized patients in China (Mao et al., 2020) to 57.4% in Europe (Romero-Sánchez et al., 2020). Among these, the following were described: headache, myalgias, confusion or agitation, impaired consciousness, stroke (Kremer et al., 2020), encephalopathy, dizziness, anosmia, dysgeusia, motor and sensory deficits, myopathy, movement disorders, ataxia, seizures (Mao et al., 2020; Romero-Sánchez et al., 2020), Guillain-Barré syndrome (Scheidl et al., 2020), acute necrotizing encephalitis (Poyiadji et al., 2020), and optic neuropathy (Romero-Sánchez et al., 2020). Altered mental status upon admission was even depicted as a predictor of in-hospital mortality (García-Azorín et al., 2021). Encephalopathy and delirium were observed more frequently in the elderly compared to younger patients and were associated with negative outcome (Kennedy et al., 2020; Liotta et al., 2020).

Considering that older patients who are more affected by neurological diseases commonly have an unpredictable clinical course, risk assessment and stratification for severity are required. Therefore, the aim of this study was to compare the clinical features and biological parameters between older (age ≥ 65 years) and younger (<65 years) patients with COVID-19 and new neurological symptoms/conditions. We also aimed to determine factors independently associated with all-cause in-hospital mortality that could serve as admission criteria and guide future therapeutic strategies.

## Materials and Methods

### Patients

We performed an observational retrospective cohort study enrolling all consecutive patients admitted in the Neurology Department of Colentina Clinical Hospital from Bucharest, Romania between 01.04 and 23.08.2020. Starting from the 16th of May 2020, Colentina Clinical Hospital has been one of the first COVID-19 support hospitals in Romania (i.e., a second line hospital that admits patients with comorbidities and SARS-CoV-2 infection in order to support the first line hospitals—infectious disease and pulmonology hospitals). Adult patients with both new neurological manifestations (including worsening of pre-existing conditions and development of novel manifestations) and SARS-CoV-2 infection (contracted before or after the emergence of neurological symptoms and confirmed by RT-PCR test for SARS-CoV-2) hospitalized in COVID-19 first line hospitals or non-COVID-19 hospitals (including emergency outpatient departments) were directed to and admitted in the Neurology Department if the severity of neurological manifestations prevailed over the course of COVID-19. Patients with a poor baseline situation requiring ICU care were directed toward other departments. All of the consecutively admitted patients in the Neurology Department during the aforementioned period were included in our study, without any exclusion criteria.

Informed consent was either signed by the patients themselves or obtained from a legal representative over the telephone (due to biosafety reasons), this fact being specified in the patient's chart. According to our local ethical regulatory items, since our clinic is affiliated to “Carol Davila” University of Medicine and Pharmacy from Bucharest, patients specifically consent for research activities when signing the informed consent upon admission—this is specified in the operational procedure regarding the access to archived data for scientific interest—PO MED 01 Edition 1. Rev 0/09.09.2015 and the operational procedure regarding the access to patient data and the processing and protection of data—PO MED 02 Edition 1. Rev 0/01.07.2019. The study was approved by the local Ethics Committee (Nr.496/10.09.2020) and was completed in conformity with the World Medical Association Declaration of Helsinki from 1975.

### Management

Management of the patients was carried out in accordance with the internal and the national treatment guidelines corresponding to the aforementioned period, which were timely updated (Ministerul Sănătăii – M. S., 2020). The patients were evaluated and monitored by the neurology team as well as an infectionist appointed to our clinic. The follow-up lasted for the period of hospitalization as it was intended to determine the prognostic factors related to in-hospital mortality.

The standard treatment included supplemental oxygen by non-invasive mechanical ventilation: nasal cannula, facial mask or high-flow ventilation according to patient's requirements; medication involved low-molecular-weight heparin in prophylactic or therapeutic dosage, dexamethasone, antibiotics (ceftriaxone, linezolid, meropenem, amoxicillin/clavulanic acid, vancomycin, azithromycin, and doxycycline), remdesivir, and tocilizumab. The scheme was adapted individually based on patient's clinical and paraclinical parameters. Some patients also received hydroxychloroquine and/or lopinavir/ritonavir at the beginning of the pandemic. Patients with severe forms and negative evolution were transferred to the ICU. During hospitalization, all patients underwent at least one lung CT scan upon admission—as an internal standard of care, or they had performed the CT in the hospital from where the patient was redirected to our institution. The severity of COVID-19 was ranked as follows: mild form for patients with blood oxygen saturation >95% who did not require oxygen supplementation and had <10% pulmonary involvement on CT scan, moderate form for patients who required oxygen supplementation by non-invasive mechanical devices and had between 10 and 50% pulmonary involvement on CT scan, and severe form for all other patients with >50% lung involvement and/or invasive mechanical ventilation.

### Laboratory Results

Blood was extracted by venipuncture in clot activator vacutainer tubes for serum separation, afterwards it was processed in a COBAS 8000 Analyzer or in a VITROS 5.1 FS for biochemistry and in a SYSMEX XN 3000 Analyzer or in a DxH 900 High Volume Lab Hematology Analyzer for complete blood count. The extraction of SARS-CoV-2 was completed with the use of an automatic extractor and certified and validated by CE-IVD Real Time PCR Kits with the CFX 96 Analyzer (GRAL Medical Molecular Biology Laboratory, Bucharest), respecting the workflow and all necessary conditions with BSL-2 safety level.

### Data Analysis

Patients were segregated into two study groups: non-elderly (including patients <65 years old) and elderly (involving subjects ≥65 years old). Patient characteristics such as demographic data, comorbidities, biological parameters, imaging findings, and clinical course were recorded. In-hospital mortality was the main outcome, while COVID-19 severity, hospitalization duration and the levels of supplemental oxygen were the secondary outcomes.

Database design and data analysis were performed using IBM SPSS Statistics 25. Categorical variables were reported as frequency and analyzed with Chi-square test. Continuous variables were reported as median (minimum, maximum). We considered parametric tests when distribution of the continuous variable was normal and non-parametric tests when distribution was not normal. We tested normality with Kolmogorov–Smirnov tests. Since all variables had non-normal distribution, Mann–Whitney *U-*tests were applied. In the logistic regression, we adjusted for all the variables that significantly correlated with the mortality (*p* < 0.05); we used age as a continuous variable. They were selected as covariates by the “enter” method, with mortality (yes/no) as a dependent variable. Hypothesis testing was two-tailed and statistical significance was defined as *p* < 0.05. A number of 140 patients were necessary to be included in order to develop a regression model with 12 predictors, at an estimated outcome proportion of 0.25 with a root mean square percentage error set at 0.14 (https://mvansmeden.shinyapps.io/BeyondEPV/). We report missing values in the following variables: comorbidity (two missing values), history of arterial hypertension, diabetes mellitus, atrial fibrillation, dyslipidemia, stroke, ischemic heart disease, chronic kidney disease and dementia (each with four missing values).

## Results

One hundred forty-eight patients were included, all Caucasians of Romanian descent. The mean age was 59.84 ± 19.06 and the majority were females (54.1%) and from urban areas (81.8%).

Demographic data, comorbidities (including risk factors for severe COVID-19 illness and neurological disorders), and neurological symptoms/manifestations upon admission are listed in Table 1. The elderly group consisted of 70 patients (47.3%), out of which 57.1% were women. Comorbidities were significantly more frequent in this group (*p* < 0.001), including neurological disorders. Apart from overweight/obesity, all other risk factors (i.e., cardiovascular and metabolic disorders) for severe COVID-19 illness significantly prevailed among the elderly.

**Table 1:** Demographic and clinical characteristics of patients.

**Characteristics**		**Non-elderly, *N* (%)**	**Elderly, *N* (%)**	***p*-value**	**OR**	**95% CI**
		**78 (52.7%)**	**70 (47.3%)**			
**Demographic factors**	Age, years (mean, range)	47 (20–64)	76 (65–93)			
	Sex, females *N* (%)	40 (51.3%)	40 (57.1%)	0.475		
	Area, urban *N* (%)	64 (82.1%)	57 (81.4%)	0.922		
**Comorbidities (risk factors for severe COVID-19 illness)**	Dyslipidemia, *N* (%)	13 (16.9%)	40 (59.7%)	<0.001	7.293	[3.37, 15.76]
	Arterial hypertension					
	Grade 1, *N* (%)	3 (3.9%)	18 (26.9%)	<0.001	26.165^*^	[10.23, 66.89]
	Grade 2, *N* (%)	9 (11.7%)	22 (32.8%)			
	Grade 3, *N* (%)	7 (9.1%)	20 (29.9%)			
	Overweight/Obesity, *N* (%)	18 (25.4%)	22 (38.6%)	0.252		
	Diabetes mellitus, *N* (%)	7 (9.1%)	25 (37.3%)	<0.001	5.952	[2.36, 14.95]
	Atrial fibrillation, *N* (%)	4 (5.2%)	29 (43.3%)	<0.001	13.927	[4.56, 42.53]
	Ischemic heart disease, *N* (%)	5 (6.5%)	24 (35.8%)	<0.001	8.037	[2.85, 22.62]
	Chronic kidney disease, *N* (%)	2 (2.6%)	11 (16.4%)	0.004	7.366	[1.56, 34.56]
**Neurological comorbidities**	Prior stroke, *N* (%)	5 (6.49%)	27 (39.13%)	<0.001	8.037	[2.85, 22.62]
	Dementia, *N* (%)	0 (0%)	11 (15.94%)	0.004	19.214	[1.07, 343.13]
	Parkinson's disease, *N* (%)	1 (1.29%)	3 (4.34%)	–		
	Epilepsy, *N* (%)	9 (11.68%)	1 (1.44%)	0.232		
	Other^**^, *N* (%)	12 (15.58%)	0 (0%)	–		
**Neurological symptoms/manifestations**	Headache, *N* (%)	31 (39.7%)	5 (7.1%)	–		
	Dizziness, *N* (%)	8 (10.3%)	1 (1.4%)	–		
	Confusion, *N* (%)	0 (0%)	8 (11.4%)	–		
	Coma, *N* (%)	3 (3.8%)	6 (8.6%)	–		
	Epileptic seizure, *N* (%)	3 (10.3%)	1 (1.4%)	–		
	Motor deficit, *N* (%)	12 (15.4%)	14 (20%)	–		
	Sensory manifestations, *N* (%)	2 (2.6%)	1 (1.4%)	–		
	Ataxia, *N* (%)	1 (%)	1 (1%)	–		
	Language impairment, *N* (%)	0 (%)	4 (%)	–		
	Hyposmia, *N* (%)	2 (2.6%)	0 (%)	–		
	Combined^***^, *N* (%)	9 (11.5%)	19 (27.1%)	–		

*OR values and 95% confidence interval (CI) were presented only for significant associations*;

* *OR is calculated for arterial hypertension presence (no/yes) within the two groups*;

** *includes: multiple sclerosis, neurodevelopmental disorders, peripheral vestibular syndrome, myasthenia gravis, history of head trauma, etc.*;

*** *includes: motor deficit+sensory manifestation, motor deficit+language impairment, etc*.

The final neurological diagnosis is presented in Table 2, with significant differences between the two groups (*p* < 0.001). In the elderly group, stroke, acute confusional state in dementia, and worsening parkinsonism in Parkinson's disease were most frequently encountered (65.71, 14.28, and 4.28%, respectively), whereas the non-elderly commonly had secondary headache (attributed to SARS-CoV-2 infection), stroke, and peripheral vestibular syndrome (38.46, 8.97, and 7.69%, respectively).

**Table 2:** Neurological diagnosis.

**Non-elderly, *N* (%)**	**Elderly, *N* (%)**
**78 (52.7%)**	**(47.3%)**
1. Secondary headache, 30 (38.46%) 2. Stroke, 15 (19.23%): - Ischemic stroke, 11 - Haemorrhagic stroke, 2 - Cerebral venous - thrombosis, 2 3. Peripheral vestibular syndrome, 7 (8.97%) 4. Multiple sclerosis relapse, 6 (7.69%) 5. Epilepsy, 5 (6.41%) 6. Encephalitis, 2 (2.56%) 7. Guillain-Barre syndrome, 1 (1.28%) 8. Worsening parkinsonism in Parkinson's disease, 1 (1.28%) 9. Myasthenic crisis, 1 (1.28%)	1. Stroke, 46 (65.71%): - Ischemic, 41 - Haemorrhagic, 4 - Cerebral venous thrombosis, 1 2. Acute confusional state in dementia, 10 (14.28%) 3. Worsening parkinsonism in Parkinson's disease, 3 (4.28%) 4. Secondary headache, 3 (4.28%) 5. Encephalitis, 1 (1.42%) 6. Hypercapnic encephalopathy, 1 (1.42%) 7. Epilepsy, 1 (1.42%)

Paraclinical characteristics (biological parameters and imaging findings) are listed in Table 3. The elderly had significantly higher median values of white blood cell (*p* = 0.02) and neutrophil count (*p* = 0.003), C-reactive protein (CRP), ferritin, fibrinogen, D-dimer (*p* < 0.001 for each), prothrombin time (PT), aspartate transaminase (AST) (*p* = 0.001 for each), creatinine, and blood urea nitrogen level (BUN) (*p* < 0.001) as well as lower median value of hemoglobin (*p* = 0.007) and lymphocyte count (*p* = 0.001). Moreover, advanced age was significantly associated with higher percentage of lung involvement (*p* < 0.001).

**Table 3:** Paraclinical characteristics of patients.

**Characteristics**	**Non-elderly, *N* (%)**	**Elderly, *N* (%)**	***p*-value**
	**78 (52.7%)**	**70 (47.3%)**	
Hemoglobin, g/dL	13.9 (8, 17.4)	13.05 (5.7, 17.8)	0.007
Leukocytes, /μL	6,690 (2,700, 26,000)	8,060 (2,100, 20,500)	0.022
Neutrophils, /μL	4,125 (1,100, 23,400)	6,060 (1,400, 18,100)	0.003
Lymphocytes, /μL	1,670 (300, 4,000)	1,245 (400, 9,800)	0.001
Platelets, × 10^3^ /μL	244.5 (47, 462)	205.5 (63, 460)	0.06
CRP, mg/L	5.72 (0.2, 222.2)	29.27 (0.3, 343.1)	<0.001
Ferritin, μg/L	187 (12.9, 4,070)	482 (23, 10,538)	<0.001
Fibrinogen, mg/dL	374 (117, 675)	477 (115, 907)	<0.001
D-dimer	0.42 (0.2, 21)	1.16 (0, 21)	<0.001
PT, s	13.8 (11.3, 26.9)	15.15 (12.2, 50.9)	0.001
aPTT, s	28.9 (7.1, 38.4)	29.5 (22.7, 53.5)	0.109
IL-6, pg/mL	28.9 (1.5, 989.6)	48.96 (5.9, 603.9)	0.293
AST, U/L	20.8 (11.2, 145.9)	26.8 (8.7, 121.8)	0.001
ALT, U/L	20.3 (2.8, 106.6)	21 (6.2, 107.9)	0.908
GGT, U/L	43 (7, 1,626)	46.5 (8, 530)	0.309
Creatinine, mg/dL	0.77 (0.2, 8.9)	0.96 (0.2, 4)	<0.001
BUN, mg/dL	27.65 (0.6–104)	51.1 (13.7–139.1)	<0.001
Sodium, mmol/L	140 (132, 149)	140 (126, 151)	0.846
Potassium, mmol/L	4.05 (3, 6.6)	3.96 (2.8, 5.8)	0.781
Pulmonary involvement, %	10 (0, 60)	25 (0, 80)	<0.001
Viral positivity duration, days	15 (0, 51)	12 (0, 67)	0.575

The clinical outcome of patients is presented in Table 4. Thirty-five patients (23.6%) died. The elderly had significantly more severe COVID-19 illness (*p* < 0.001), higher need for supplemental oxygen (*p* < 0.001), and higher mortality rate than the non-elderly (*p* < 0.001).

**Table 4:** Clinical outcome of patients.

**Characteristics**	**Non-elderly, *N* (%)**	**Elderly, *N* (%)**	***p*-value**	**OR**	**95% CI**
	**78 (52.7%)**	**70 (47.3%)**			
Hospitalization duration, days	14 (1, 70)	12.5 (1, 140)	0.897		
Supplemental	0 (0, 8)	0 (0, 10)	<0.001		
oxygen, L/min	2 MV	11 MV			
COVID-19 severity					
Mild, *N* (%)	43 (55.12%)	25 (35.71%)			
Moderate, *N* (%)	28 (35.89%)	33 (47.14%)	<0.001		
Severe, *N* (%)	7 (8.97%)	12 (17.14%)			
Mortality, *N* (%)	7 (9%)	28 (40%)	<0.001	6.761	[2.71, 16.83]

*OR values and 95% confidence interval (CI) were presented only for significant associations; MV=mechanical ventilation*.

In the univariate analysis, apart from advanced aged, mortality was significantly associated with comorbidity presence, initial hemoglobin level, neutrophil and lymphocyte count, inflammatory markers, coagulation tests, AST, and BUN (Table 5).

**Table 5:** Factors associated with mortality in the univariate analysis.

**Characteristics**	**Survivor, *N* (%)**	**Non-survivor, *N* (%)**	***p*-value**	**OR**	**95% CI**
	**113 (76.4%)**	**35 (23.6%)**			
Age, years (mean, range)	55 (20, 85)	79 (39–93)	<0.001		
Comorbidity (no/yes), *N* (%)	33/79 (29.46/70.53%)	0/34 (0/100%)	<0.001	29.075	[1.73, 488.24]
Hemoglobin, g/dL	13.8 (7.9, 17.8)	12.6 (5.7, 16.6)	0.013		
Neutrophils, /μL	4.18 (1.1, 18.1)	8.13 (2.8, 23.4)	<0.001		
Lymphocytes, /μL	1.6 (0.4, 9.8)	1.05 (0.3, 2.8)	<0.001		
CRP, mg/L	7.53 (0.2, 343.1)	88.11 (0.4, 279.2)	<0.001		
Ferritin, μg/L	251.5 (12.9, 3,115)	703.5 (59.2, 10,538)	<0.001		
Fibrinogen, mg/dL	398 (115, 907)	490 (117, 807)	0.002		
D-dimer, μg/ml FEU	0.5 (0, 21)	1.81 (0.3, 21)	<0.001		
PT, s	14 (11.3, 50.9)	16.2 (12.6, 43.3)	<0.001		
aPTT, s	28.9 (7.1, 53.5)	32.1 (22.7, 48.9)	0.004		
AST, U/L	22.2 (11.2, 145.9)	33 (8.7, 107.8)	0.005		
BUN, mg/dL	32.3 (0.6, 102.9)	51.1 (18.1, 139.1)	<0.001		

*OR values and 95% confidence interval (CI) were presented only for significant associations*.

After adjusting for all these possible confounders, advanced age remained associated with mortality. In addition to age, neutrophil count, CRP, fibrinogen, and aPTT levels also remained associated with fatality rate (Table 6). Interestingly, comorbidity presence did not reach statistical significance.

**Table 6:** Age as predictor of mortality, adjusted for blood cell count, biological inflammatory markers, coagulation tests, AST, and BUN (logistic regression).

**Variables**	**B**	**OR**	**95% CI for OR**		***p*-value**
			**Lower**	**Upper**	
Age	0.107	1.113	1.039	1.191	0.002
Hemoglobin	0.142	1.153	0.803	1.654	0.440
Neutrophils	0.281	1.324	1.043	1.682	0.021
Lymphocytes	−0.422	0.656	0.147	2.920	0.580
CRP	0.015	1.015	1.000	1.030	0.049
Ferritin	0.001	1.001	1.000	1.002	0.064
Fibrinogen	−0.009	0.991	0.985	0.998	0.011
D-dimer	−0.055	1.008	0.811	1.104	0.484
PT	−0.034	0.946	0.858	1.071	0.452
aPTT	0.189	1.225	1.033	1.452	0.020
AST	−0.005	0.995	0.962	1.030	0.785
BUN	0.000	1.000	0.966	1.0366	0.987
Comorbidity	17.043	25,228,154.49			0.998

## Discussion

The results of this observational retrospective cohort study revealed that elderly patients (age ≥ 65 years) with new neurological symptoms have poorer outcomes than younger ones. A possible explanation is that older age was significantly associated with more extensive lung involvement, lower oxygen saturation levels, and higher need for oxygen supplementation. Consequently, these patients had more severe COVID-19 (17.47%) and required mechanical ventilation and ICU admission more often (8.97%) than younger patients, as it was stated in previous studies (Guo et al., 2020; Lian et al., 2020). In line with other publications, advanced age was significantly associated with: leucocytosis, neutrophilia, lymphocytopenia, anemia, a more severe inflammatory syndrome reflected by higher values of CRP, fibrinogen and ferritin, increased levels of D-dimers, as well as impaired renal and hepatic function (based on increased values of BUN, creatinine, AST and PT) (Kennedy et al., 2020). Therefore, the mortality rate (23.6%) was significantly associated with advanced age (*p* < 0.001), as it was stated in previous reports Centers for Disease Control and (ISS (Istituto Superiore di Sanità)., 2020; Salje et al., 2020; Wu and McGoogan, 2020). Mortality was 4.4 times more common in elderly patients than in non-elderly ones, an approximatively similar rate to another publication (Giangreco, 2020). Moreover, after adjusting for other possible confounders apart from age, mortality was significantly associated with higher neutrophil count, fibrinogen, and aPTT levels. However, in contrast to other results (Karlberg et al., 2004), mortality did not correlate with gender.

According to other studies, elderly patients tend to have slightly different symptoms upon admission; they often complain of fatigue, myalgia, digestive symptoms, headache (Guo et al., 2020), behavioral changes, confusion, delirium, balance problems or even falls before the onset of respiratory symptoms (Godaert et al., 2020; Lithander et al., 2020; Neerland et al., 2020; Tay and Harwood, 2020). None of our elderly patients had digestive symptoms at presentation, but confusion was the only initial symptom in 14.28% of them, mostly in patients with dementia. Since hypoxia could potentially complicate the presentation of dementia and promote delirium (Marcantonio, 2017) [which is associated with high risk for negative outcomes, such as ICU stay, the need to be discharged to a rehabilitation institution, and mortality (Kennedy et al., 2020)], we advocate for hospitalizing elderly patients with confusion. Moreover, it was already ascertained that dementia is an independent risk factor for mortality (Li et al., 2020).

We emphasize the fact that the initial symptoms most commonly encountered in our patients were due to stroke in both groups, twice more frequent in the elderly. This could be explained by the fact that the elderly had at least two comorbidities in 90% of cases, out of which cardiovascular and metabolic disorders prevailed, which are predisposing risk factors for acute cerebrovascular events. In line with this, according to previous studies, the presence of such comorbidities in the elderly is linked to a higher risk of progression to a severe or even critical form of COVID-19 (Guo et al., 2020; Press et al., 2020; Sardu et al., 2020). Furthermore, according to a retrospective analysis, patients with COVID-19 and history of stroke seem to be more prone to develop ARDS, to require non-invasive and mechanical ventilation and to be transferred to the ICU; they have a poor outcome with decreased rates of discharge and increased mortality risk (Fu et al., 2020). This could be related to the anti-inflammatory responses that are generated after the occurrence of stroke, which promote infection (Emsley et al., 2008). Considering our results and previous publications (Zhai et al., 2020), it seems that severe neurological complications (e.g., stroke, acute functional decline, and delirium) are more frequent and more damaging in the elderly patients with COVID-19. Therefore, acute severe neurological conditions should be viewed as potential aggravating factors, as also stated in a previous publication (Salahuddin et al., 2020). Even in the absence of extensive pulmonary involvement, these patients require close monitoring.

### Other Possible Risk Factors for Severe COVID-19 Course in the Elderly

Apart from the fact that elderly people are possibly less compliant than younger subjects to follow authorities' recommendations such as wearing a face mask or social distancing (Daoust, 2020), the risk of developing a severe form increases with the number of comorbidities (Fu et al., 2020). However, according to a study group analysis, frailty rather than age or comorbidities may be a better predictor of poor outcome (Hewitt et al., 2020). Due to frailty, a higher proportion of old patients develop more often adverse effects related to medication and the risk-benefit might not be justifiable in selected cases such as methylprednisolone/dexamethasone use (Rosenberg et al., 2020). In addition, other possible explanations for higher prevalence of SARS-CoV-2 infection and more severe course of disease among elderly might be: age-related deterioration in the clearance of inhaled particles especially in the territory of small airways (Svartengren et al., 2005) probably due to a progressive reduction in the number of cilia and ciliated cells (Levitzky, 1984), decrease in the upper airway size which tends to be more collapsible in men, gradual increase in the volume of the nasal cavity with subsequent reduced nasal resistance with age (Xu et al., 2019) as well as reduced levels of angiotensin-converting enzyme 2 (AlGhatrif et al., 2020).

Furthermore, in regard to immune changes in the elderly, a disruption of the innate and adaptive immune system was observed, resulting in an extensive production of cytokines and inflammatory mediators—the so-called inflammaging process (Aw et al., 2007)—as well as a more profound depletion of CD4^+^ cells (Napoli et al., 2020a) that consequently lead to a disproportionate cytokine storm (Napoli et al., 2020b) and a reduced virus clearance. Also, elderly patients tend to have a vascular pro-inflammatory state, due to a reduced capacity of the senescent macrophages to phagocytose apoptotic cells (Napoli et al., 2020b). No less important is the fact that, apart from the cytokine storm and heart failure, brain injuries could also increase the mortality in older COVID-19 patients (Kremer et al., 2020).

### Limitations

The main limitations of this study originate from the small number of participants, the absence of a control group of patients without neurological conditions (in order to establish if new neurological manifestations are independent predictors of outcome) and the insufficient data in patients' charts regarding some paraclinical parameters. Other issues arise from the demographically homogenous group of patients (which might lead to difficulties in extrapolating the results to other population) and the COVID-19 severity classification that we used, which was adjusted in compliance with the local protocol (that might limit the comparison of our results with other studies). Larger cohort studies focusing on the elderly patients with new neurological conditions are required in order to establish the best approach toward them.

## Conclusions

According to our study, older age along with neutrophil count, CRP, fibrinogen, and aPTT levels are independent predictors of mortality in COVID-19 patients with new neurological manifestations at presentation. Consequently, the presence of a new neurological condition could be regarded as a risk factor for a negative outcome in the elderly patients infected with SARS-CoV-2. Inflammation markers and the extension of pulmonary involvement are important means of monitoring the severity of SARS-CoV-2 infection.

## Data Availability Statement

The raw data supporting the conclusions of this article will be made available by the authors, without undue reservation.

## Ethics Statement

The studies involving human participants were reviewed and approved by Ethical Committee—Colentina Clinical Hospital, Bucharest, Romania. The patients/participants provided their written informed consent to participate in this study.

## Author Contributions

EID, DFM, and BOP: conceptualization. EID, IO, DT, PB, and BOP: methodology. IO, DT, CDS, TB, and GS: formal analysis. EID, IO, DT, CDS, TB, and GS: investigation. EID and BOP: resources. EID, IO, and DT: writing—original draft preparation. EID, IO, PB, and BOP: writing. EID, DFM and BOP: visualization. DFM and BOP: supervision. BOP: project administration. All authors have read and agreed to the published version of the manuscript.

## Conflict of Interest

The authors declare that the research was conducted in the absence of any commercial or financial relationships that could be construed as a potential conflict of interest.

## References

[B1] AlGhatrif M. Cingolani O. Lakatta E. G. (2020). The dilemma of coronavirus disease 2019, aging, and cardiovascular disease: insights from cardiovascular aging science. JAMA Cardiol. 5, 747–748. 10.1001/jamacardio.2020.1329 32242886 PMC10089230

[B2] Aw D. Silva A. B. Palmer D. B. (2007). Immunosenescence: emerging challenges for an ageing population. Immunology 120, 435–446. 10.1111/j.1365-2567.2007.02555.x 17313487 PMC2265901

[B3] Centers for Disease Control Prevention. (2020). Severe Outcomes Among Patients with Coronavirus Disease 2019 (COVID-19)-United States, February 12-March 16, 2020. Available online at: https://www.cdc.gov/mmwr/volumes/69/wr/%0Amm6912e2.htm (accessed September 15, 2020).

[B4] Daoust J.-F. (2020). Elderly people and responses to COVID-19 in 27 Countries. PLoS One. 15:e0235590. 10.1371/journal.pone.0235590 32614889 PMC7332014

[B5] Demombynes G. (2020). COVID-19 Age-Mortality Curves Are Flatter in Developing Countries. Policy Research Working Paper. Report No.: 9313. Washington, DC. Available online at: http://hdl.handle.net/10986/34028 (accessed March 30, 2021).

[B6] Deutsche Akademie der Naturforscher Leopoldina e.V. (2020). Nationale Akademie der Wissenschaften. Available online at: http://www.leopoldina.org (accessed September 15, 2020).

[B7] Emsley H. C. A. Smith C. J. Hopkins S. J. (2008). Infection and brain-induced immunodepression after acute ischemic stroke. Stroke 39:e7; author reply e8. 10.1161/STROKEAHA.107.500447 18048842

[B8] Excess Mortality. (2020a). Excess Mortality in EU. Available online at: https://www.euromomo.eu/graphs-and-maps/#excess-mortality (accessed March 29, 2021).

[B9] Excess Mortality. (2020b). Excess Mortality in US. Available online at: https://www.cdc.gov/mmwr/volumes/69/wr/mm6942e2.htm (accessed March 29, 2021).

[B10] Fu L. Wang B. Yuan T. Chen X. Ao Y. Fitzpatrick T.. (2020). Clinical characteristics of coronavirus disease 2019 (COVID-19) in China: a systematic review and meta-analysis. J. Infect. 80, 656–665. 10.1016/j.jinf.2020.03.041 32283155 PMC7151416

[B11] García-Azorín D. Trigo J. Martínez-Pías E. Hernández-Pérez I. Valle-Peñacoba G. Talavera B.. (2021). Neurological symptoms in Covid-19 patients in the emergency department. Brain Behav. 11:e02058. 10.1002/BRB3.2058/v2/response1 33617117 PMC7994975

[B12] Giangreco G. (2020). Case fatality rate analysis of Italian COVID-19 outbreak. J. Med. Virol. 92, 919–923. 10.1002/jmv.25894 32297983 PMC7262321

[B13] Godaert L. Proye E. Demoustier-Tampere D. Coulibaly P. S. Hequet F. Dramé M. (2020). Clinical characteristics of older patients: the experience of a geriatric short-stay unit dedicated to patients with COVID-19 in France. J. Infect. 81, e93–e94. 10.1016/j.jinf.2020.04.009 32305489 PMC7162788

[B14] Guo T. Shen Q. Guo W. He W. Li J. Zhang Y.. (2020). Clinical characteristics of elderly patients with COVID-19 in Hunan Province, China: a multicenter, retrospective study. Gerontology 66, 467–475. 10.1159/000508734 32474561

[B15] Hewitt J. Carter B. Vilches-Moraga A. Quinn T. J. Braude P. Verduri A.. (2020). The effect of frailty on survival in patients with COVID-19 (COPE): a multicentre, European, observational cohort study. Lancet Public Health 5, e444–e451. 10.1016/S2468-2667(20)30146-8 32619408 PMC7326416

[B16] Institutul National de Sănătate Publică (2020). Covid 19 Raport Săptămânal de Supraveghere. Available online at: http://www.cnscbt.ro/index.php/analiza-cazuri-confirmate-covid19/1965-raport-saptamanal-episaptamana35/file (accessed September 15, 2020).

[B17] ISS (Istituto Superiore di Sanità). (2020). Available online at: https://www.epicentro.iss.it/%0Acoronavirus/bollettino/Bollettino-sorveglianza-integrata-COVID-19_28-%0Aaprile-2020.pdf (accessed September 15, 2020).

[B18] Karlberg J. Chong D. S. Y. Lai W. Y. Y. (2004). Do men have a higher case fatality rate of severe acute respiratory syndrome than women do? Am. J. Epidemiol. 159, 229–231. 10.1093/aje/kwh056 14742282 PMC7110237

[B19] Kennedy M. Helfand B. K. I. Gou R. Y. Gartaganis S. L. Webb M. Moccia J. M.. (2020). Delirium in older patients with COVID-19 presenting to the emergency department. JAMA Netw. Open 3:e2029540. 10.1001/jamanetworkopen.2020.29540 33211114 PMC7677760

[B20] Kremer S. Lersy F. Anheim M. Merdji H. Schenck M. Oesterlé H.. (2020). Neurologic and neuroimaging findings in patients with COVID-19: a retrospective multicenter study. Neurology 95:e1868–e1882. 10.1212/WNL.0000000000010112 32680942

[B21] Levitzky M. G. (1984). Effects of aging on the respiratory system. Physiologist 27, 102–107. 6374697

[B22] Li P. Chen L. Liu Z. Pan J. Zhou D. Wang H.. (2020). Clinical features and short-term outcomes of elderly patients with COVID-19. Int. J. Infect. Dis. 97, 245–250. 10.1016/j.ijid.2020.05.107 32492533 PMC7261456

[B23] Lian J. Jin X. Hao S. Cai H. Zhang S. Zheng L.. (2020). Analysis of epidemiological and clinical features in older patients with coronavirus disease 2019 (COVID-19) outside Wuhan. Clin. Infect. Dis. 71, 740–747. 10.1093/cid/ciaa242 32211844 PMC7184356

[B24] Liotta E. M. Batra A. Clark J. R. Shlobin N. A. Hoffman S. C. Orban Z. S.. (2020). Frequent neurologic manifestations and encephalopathy-associated morbidity in Covid-19 patients. Ann. Clin. Transl. Neurol. 7, 2221–2230. 10.1002/acn3.51210 33016619 PMC7664279

[B25] Lithander F. E. Neumann S. Tenison E. Lloyd K. Welsh T. J. Rodrigues J. C. L.. (2020). COVID-19 in older people: a rapid clinical review. Age Ageing 49, 501–515. 10.1093/ageing/afaa093 32377677 PMC7239238

[B26] Mao L. Jin H. Wang M. Hu Y. Chen S. He Q.. (2020). Neurologic manifestations of hospitalized patients with coronavirus disease 2019 in Wuhan, China. JAMA Neurol. 77, 683–690. 10.1001/jamaneurol.2020.1127 32275288 PMC7149362

[B27] Marcantonio E. R. (2017). Delirium in hospitalized older adults. N. Engl. J. Med. 377, 1456–1466. 10.1056/NEJMcp1605501 29020579 PMC5706782

[B28] Ministerul Sănătăii – M. S. (2020). Protocolul de tratament al infec?iei cu virusul SARS-Cov-2, din 26.03.2020. Romania. Available online at: http://www.cnscbt.ro/index.php/lex/1617-protocolul-de-tratament-covid-19/file (accessed September 15, 2020).

[B29] Napoli C. Tritto I. Benincasa G. Mansueto G. Ambrosio G. (2020b). Cardiovascular involvement during COVID-19 and clinical implications in elderly patients. A review. Ann. Med. Surg. 57, 236–243. 10.1016/j.amsu.2020.07.054 32802325 PMC7403130

[B30] Napoli C. Tritto I. Mansueto G. Coscioni E. Ambrosio G. (2020a). Immunosenescence exacerbates the COVID-19. Arch. Gerontol. Geriatr. 90:104174. 10.1016/j.archger.2020.104174 32653765 PMC7333612

[B31] Neerland B. E. Dobloug A. Nore K. G. Mikaelsen E. E. Halsen A. Ahmed M. V. (2020). COVID-19 in an elderly woman with acute functional decline. Tidsskr. Nor. Laegeforen. 140, 1–4. 10.4045/tidsskr.20.0307 32378845

[B32] Poyiadji N. Shahin G. Noujaim D. Stone M. Patel S. Griffith B. (2020). COVID-19-associated acute hemorrhagic necrotizing encephalopathy: imaging features. Radiology 296, E119–E120. 10.1148/radiol.2020201187 32228363 PMC7233386

[B33] Press V. G. Gershon A. S. F Sciurba F. C. Blagev D. P. (2020). Concerns about COVID-related collateral damage for patients with COPD. Chest 158, 866–868. 10.1016/j.chest.2020.05.549 32473947 PMC7837125

[B34] Romero-Sánchez C. M. Díaz-Maroto I. Fernández-Díaz E. Sánchez-Larsen Á. Layos-Romero A. García-García J.. (2020). Neurologic manifestations in hospitalized patients with COVID-19: the ALBACOVID registry. Neurology 95, e1060–e1070. 10.1212/WNL.0000000000009937 32482845 PMC7668545

[B35] Rosenberg E. S. Dufort E. M. Udo T. Wilberschied L. A. Kumar J. Tesoriero J.. (2020). Association of treatment with hydroxychloroquine or azithromycin with in-hospital mortality in patients with COVID-19 in New York State. JAMA 323, 2493–2502. 10.1001/jama.2020.8630 32392282 PMC7215635

[B36] Salahuddin H. Afreen E. Sheikh I. S. Lateef S. Dawod G. Daboul J.. (2020). Neurological predictors of clinical outcomes in hospitalized patients with COVID-19. Front. Neurol. 11:585944. 10.3389/fneur.2020.585944 33193048 PMC7662675

[B37] Salje H. Tran Kiem C. Lefrancq N. Courtejoie N. Bosetti P. Paireau J.. (2020). Estimating the burden of SARS-CoV-2 in France. medRxiv 369, 208–211. 10.1101/2020.04.20.20072413 PMC7223792 32404476

[B38] Sardu C. Gambardella J. Morelli M. B. Wang X. Marfella R. Santulli G. (2020). Hypertension, thrombosis, kidney failure, and diabetes: is COVID-19 an endothelial disease? A comprehensive evaluation of clinical and basic evidence. J. Clin. Med. 9:1417. 10.3390/jcm9051417 32403217 PMC7290769

[B39] Scheidl E. Canseco D. D. Hadji-Naumov A. Bereznai B. (2020). Guillain-Barré syndrome during SARS-CoV-2 pandemic: a case report and review of recent literature. J. Peripher. Nerv. Syst. 25, 204–207. 10.1111/jns.12382 32388880 PMC7273104

[B40] Svartengren M. Falk R. Philipson K. (2005). Long-term clearance from small airways decreases with age. Eur. Respir. J. 26, 609–615. 10.1183/09031936.05.00002105 16204590

[B41] Tay H. S. Harwood R. (2020). Atypical presentation of COVID-19 in a frail older person. Age Ageing 49, 523–524. 10.1093/ageing/afaa068 32315386 PMC7188159

[B42] Wu Z. McGoogan J. M. (2020). Characteristics of and important lessons from the coronavirus disease 2019 (COVID-19) outbreak in China: summary of a report of 72,314 cases from the Chinese Center for Disease Control and Prevention. JAMA 323, 1239–1242. 10.1001/jama.2020.2648 32091533

[B43] Xu J. Kang Y.-A. Park S.-K. Yoon Y. H. Bai S. J. Jin Y.. (2019). Nasality changes with age in normal Korean-speaking adults. Clin. Exp. Otorhinolaryngol. 12, 95–99. 10.21053/ceo.2018.00717 30257547 PMC6315219

[B44] Zhai P. Ding Y. Li Y. (2020). The impact of COVID-19 on ischemic stroke. Diagn. Pathol. 15:78. 10.1186/s13000-020-00994-0 32600350 PMC7323364

